# A Bio-Inspired Antigen-Antibody Optimization Approach for MRI-Based Stroke Lesion Segmentation

**DOI:** 10.2174/0115734056482692260730115330

**Published:** 2026-08-04

**Authors:** S. Sylvia Irish, J. Dhalia Sweetlin, P. Horsley Solomon

**Affiliations:** 1 Department of Information Technology, Anna University, MIT Campus, Chennai, India; 2 Department of Electronics with Artificial Intelligence, SRM Arts and Science College, Chennai, India

**Keywords:** Ischemic stroke, superpixel segmentation, MRI, Cellular automata, Antigen-antibody binding, Simple linear iterative clustering

## Abstract

**Objective:**

Ischemic strokes constitute a significant global health issue, contributing to high death rates and chronic disability, which creates an urgent need for precise and efficient detection methods. Accurate segmentation and classification of ischemic stroke lesions from magnetic resonance imaging (MRI) remains a challenging task due to lesion heterogeneity, intensity variations, and limited annotated datasets. This study investigates the feasibility of integrating an antigen-antibody-inspired optimization strategy into a lesion segmentation framework for ischemic stroke MRI analysis.

**Methods:**

The proposed proof-of-concept framework combines Cellular Automata (CA)-based brain masking, Simple Linear Iterative Clustering (SLIC) based superpixel segmentation, and an antigen-antibody-inspired optimization mechanism for segmentation refinement. The optimized lesion regions are subsequently analyzed using a ResNet-50 deep learning model for feature extraction and classification. MRI data from the ISLES-2015 dataset were used for methodological evaluation under heterogeneous lesion conditions.

**Results:**

The proposed framework achieved a classification accuracy of 93.09%, pixel accuracy of 96.96%, precision of 0.723, recall of 0.76, F1-score of 0.71, Intersection over Union (IoU) of 0.56, and Dice coefficient of 0.71.

**Discussion:**

The antigen-antibody-inspired optimization stage has contributed to improved lesion-region refinement compared with baseline segmentation output, thereby improving the classification results. These findings indicate that biologically inspired optimization can effectively complement conventional image segmentation techniques in handling ischemic stroke lesions.

**Conclusion:**

The proposed framework effectively utilizes biologically inspired optimization strategies for segmentation refinement in ischemic stroke MRI analysis. However, the present work should be interpreted as an exploratory methodological proof-of-concept rather than a clinically validated diagnostic framework. Further evaluation using clinically representative datasets, external validation cohorts, and multicenter studies is required to establish robustness and clinical applicability.

## 1 INTRODUCTION

A stroke is a brain disorder that occurs due to a blockage in the blood vessels. It happens due to a blood clot in the brain that obstructs the flow of blood or due to the rupturing blood vessels, which in turn leads to bleeding [1]. About 87% of all strokes are ischemic strokes, which are the prevalent form of brain stroke and cause significant death and long-term impairment. Ischemic stroke continues to be a major worldwide health concern that affects people and communities, making it a major challenge to global healthcare. An ischemic stroke develops when a clot or buildup of plaque obstructs blood flow to the brain. This causes serious consequences if treatment is delayed [2].

Effective and early intervention requires faster diagnosis, as postponing therapy might cause significant brain damage and permanent impairments. Understanding the severity and urgency of ischemic strokes with the aim of improving detection accuracy using different imaging techniques helps in the efficient treatment of stroke. The two types of neuroimaging techniques are structural and functional [3].

Early recognition of ischemic stroke remains a critical clinical challenge, particularly during the initial hyperacute stages when neurological symptoms may be subtle and neuroimaging findings are often inconclusive [4]. In routine clinical practice, early stroke identification relies not only on neuroimaging but also on the analysis of neurological deficits, including facial asymmetry, limb weakness, speech disturbances, and cognitive impairment. Clinical assessment serves as the initial point of evaluation for healthcare professionals and involves comprehensive evaluation of medical history, risk factors, and physical examination [4]. The NIHSS is a standardized clinical assessment tool used to measure stroke severity by evaluating neurological functions such as consciousness, motor ability, sensation, language, and vision [5]. It helps clinicians assess stroke severity, monitor progression, and guide treatment decisions. Consequently, imaging-based computational approaches should be viewed as complementary tools that assist lesion identification and characterization alongside established clinical assessment procedures [5].

Structural imaging aims to focus on visualizing anatomical structures and their corresponding abnormalities in the brain, while functional imaging evaluates brain activity across different regions. Structural neuroimaging encompasses techniques like Computed Tomography (CT) and Magnetic Resonance Imaging (MRI), while functional neuroimaging includes methods such as functional MRI (fMRI) and Positron Emission Tomography (PET). Because CT is readily available, inexpensive, and takes less time to image, it is a particularly common neuroimaging method. Further, CT and MRI imaging produce high-resolution images of the body’s bony structures and soft tissues, respectively. Additionally, compared to CT, MRI offers superior contrast between normal brain tissues, lesion tissues, muscles, connective tissues, *etc*. [6].

The proposed work offers a novel solution by combining methods such as the antibody-antigen algorithm [7] for accurate stroke zone delineation, superpixel segmentation using the Simple Linear Iterative Clustering algorithm [8], and Cellular Automata [9] for brain mask extraction. Further, the deep learning algorithm uses neural networks like the Residual Network (ResNet) for intelligent pattern identification and predictive analysis [10]. This approach enables the investigation of biologically inspired optimization strategies for the refinement of ischemic stroke lesion segmentation in MRI datasets.

Cellular Automata (CA) are a computational model governed by a predefined set of rules. It uses a grid-like framework consisting of rows and columns. Each cell in CA has a finite number of states, namely, on and off. Each state of the cell is updated in accordance with a predetermined set of rules that take into account both its own and the current states of its neighbouring cells [11]. This behaviour of CA updates all cells simultaneously in the image according to the rules [11, 12]. Thus, CA enables the intrinsic patterns through the interactions and transitions of individual cells in the grid, which is used in stroke lesion identification.

Superpixels are a group of pixels with similar attributes that form meaningful regions. Common algorithms like Simple Linear Iterative Clustering (SLIC) [8] and Quickshift efficiently generate superpixels [13]. SLIC is inspired by k-means clustering, which partitions the given image into compact, uniform regions. In brain segmentation, superpixel algorithms aid in region-of-interest delineation, enhancing the subsequent processing steps [8]. The ability of the SLIC algorithm to obtain ROI enables in efficiently extract the features from the image and identify the stroke lesions.

Antibodies are the main players in the human immune response and can recognize different antigens corresponding to a particular disease. The immune response produces specific antibodies against the specific antigens, and they bind together with high affinity. For example, if the immune response produces antibody A for antigen B, then A and B will bind with high affinity. The size of the binding region between the antigen and the antibody determines the affinity strength [14]. This binding affinity between antigen and antibody aids in providing an optimized Region of Interest (ROI) for the stroke lesions.

When processing medical images, machine learning (ML) and deep learning (DL) algorithms have shown impressive outcomes. The primary benefit of ML and DL is their ability to eliminate manual feature extraction and automatically learn and extract high-level features from the data [15]. Various algorithms are used in stroke lesion segmentation [16].

Computer-Aided Diagnosis (CAD) provides improved diagnostic accuracy in image interpretation. CAD systems, including X-ray, CT, and MRI, aid radiologists and healthcare professionals in detecting and classifying abnormalities. Further, using bio-inspired computation in image segmentation enables providing optimized results [17-18].

The primary objective of this work is to investigate the feasibility of incorporating antigen antibody inspired optimization mechanisms into lesion segmentation pipelines. Accordingly, the proposed framework is presented as a proof-of-concept study evaluating the potential utility of biologically inspired refinement strategies for ischemic stroke MRI analysis.

The manuscript is organized to first review the relevant literature and highlight the advantages of the proposed approach. This is followed by a detailed description of the methodology and the working of each module. Subsequently, the experimental results are presented to evaluate the performance of the proposed method, and finally, the study concludes with a summary of the key findings.

## 2 LITERATURE SURVEY

This section discusses the presently used methods for segmenting and classification of brain stroke lesions.

Copen *et al.* provide an insight into the significance of MR perfusion imaging in acute ischemic stroke that identifies the ischemic lesions [19]. MR perfusion imaging is crucial for identifying ischemic regions by distinguishing between ischemic core with reduced blood flow and potentially salvageable penumbra tissue. It analyzes key perfusion parameters like Cerebral Blood Flow (CBF), Cerebral Blood Volume (CBV), Mean Transit Time (MTT), and time of maximum value of residue (Tmax) using imaging methods like CT and MR perfusion imaging. MR imaging is effective in the diagnosis of ischemic stroke as the imaging enables identifying the infarct core effectively.

Feng *et al.* proposed a technique for improving acute ischemic stroke diagnosis by fusing Generative Adversarial Networks (GANs) and radiomics [20]. The generated synthetic MRI-like images from CT scans provide information relevant to acute ischemic stroke. The synthetic MRI-like images were evaluated and validated against real MRI scans to provide a Mean Absolute Error (MAE) of 0.034, Peak Signal-To-Noise Ratio (PSNR) of 70.803, structural symmetry index measure (SSIM) of 0.783, Pearson Correlation Coefficient (PCC) of 0.986, and dice score of 0.869.

Tomasetti *et al.* suggested concentrating on the integration of CT perfusion (CTP) and machine learning to precisely segment hypoperfused regions [21]. This efficiently discerns the infarct core and penumbra areas, which are vital for evaluating stroke severity. In order to attain better outcomes, a thorough evaluation of several segmentation techniques, such as thresholding and machine learning, is carried out. Further, a novel multi-classification strategy based on the Random Forest (RF) algorithm is introduced that enhances the results with a Dice coefficient of 0.68 for penumbra and 0.26 for core regions.

Akter *et al.* used a machine learning approach that examines medical data in order to find patterns to identify brain stroke illness [22]. This method is used to detect and assess the severity of the ailment. Brain stroke is detected *via* classifiers including Random Forest (RF), Support Vector Machine (SVM), and Decision Tree (DT). Random Forest classifier provided an accuracy of 95.30%, a sensitivity of 95.57%, and a specificity of 25%.

Cui *et al.* proposed that Deep Learning (DL) models offer significant advantages in interpreting non-contrast CT and MRI, which are crucial in the management of ischemic stroke [23]. DL models enables in providing diagnostic results in a shorter duration with accuracy.

Yang *et al.* used a method to automatically segregate ischemic stroke lesions using an encoder-decoder convolutional neural network called IS-Net on Non-contrast CT (NCCT) images [24]. A hierarchical network and a Pyramid Feature Aggregation (PFA) module are used to aggregate features from the backbone stages. Multilevel propagation is improved, and a feature fusion approach is added. An edge constraint is also introduced to broadcast low-level features to various modules in order to completely mimic the boundary cues. The IS-Net provides a Dice score of 67.5 ± 0.9, IoU of 56.7 ± 0.5, Sensitivity of 74.4 ±1.4 and specificity of 99.1± 0.07.

Aganj *et al.* proposed a technique for soft segmentation of medical images with the use of multiple atlases to improve the segmentation accuracy [25]. The method employs a multi-atlas strategy that uses information from several pre-labeled anatomical atlases. Image segmentation using atlas-to-image transformations predicts label values at each voxel. A Fourier transform convolution produces an expected label value (ELV) map for new images, validated on benchmark datasets. The median Dice score for brain images is 0.782, with a mean of 0.766 ±0.001.

Long *et al.* presented a Fully Convolutional Network (FCN) for end-to-end pixel-level image segmentation, with FCN-8 enhancing spatial localization using skip connections to merge low- and high-level feature maps [26].

Omarov *et al.* proposed a 3D U-Net framework to enhance ischemic stroke lesion segmentation using MRI data [27]. The model used 3D convolutions to capture volumetric and contextual features across slices, improving lesion localization accuracy. It provided a Dice score of 0.58 in training with precision and recall of 0.68 and 0.60, respectively.

Rao *et al.* proposed a predictive model for stroke disease that utilizes CNNs and incorporates information from multiple biosignals [28]. The multimodal biosignals used are electrocardiography (ECG), electroencephalography (EEG), blood pressure, and other relevant biosensors. The multimodal approach enhances understanding of a patient’s physiological state. A CNN extracts key features from multimodal biosignals, identifying patterns linked to stroke risk. Standard metrics like sensitivity, specificity, accuracy, and AUC-ROC assess the CNN model’s performance, which achieved an accuracy of 99.15% for the CNN-LSTM model.

Hui *et al.* suggested a novel segmentation approach that involves a partitioning-stacking prediction fusion network that is built upon an enhanced Attention U-Net for the specific task of stroke lesion segmentation [29]. The proposed model likely incorporates partitioning and stacking strategies to fuse predictions effectively. The Attention U-Net, known for its ability to capture intricate image features, is used to improve and enhance stroke lesion segmentation accuracy. The partitioning strategy divides input data into segments, while the stacking strategy aggregates predictions to enhance segmentation results. The method showed a Dice coefficient of 0.593.

Ho *et al.* presents a ML approach for classifying the inception time of ischemic strokes based on imaging data [30]. Advanced techniques in machine learning accurately categorize the time of onset for ischemic strokes, which is a critical factor in determining treatment strategies and outcomes. The classification involves analyzing features from imaging data like MRI and CT scans and training a machine learning model with a dataset annotated for onset times. The best classifier demonstrated superior performance compared to previous techniques, achieving a cross-validated area under the curve of 0.765, a sensitivity of 0.788, and a negative predictive value of 0.609.

Lui *et al.* suggested using a hybrid contextual semantic network (HCSNet) for accurately segmenting and identifying small-sized stroke lesions from MRI data [31]. To create semantic features, it employs a hybrid contextual semantic module and an encoder-decoder architecture. The tiny lesions are optimized using a mixing loss function. The HCSNet provides a DC of 69.72, ASSD of 9.24, precision of 75.86, and recall of 66.93.

Gheibi *et al.* used a deep convolutional neural network to segment ischemic strokes from multimodal MRIs [32]. The method uses encryption-decryption paths with residual networks. The model provided a Dice coefficient of 85.43% on a dataset obtained from the Neuroscience Center of Tabriz University of Medical Sciences and a 79.23% Dice coefficient on the SPES 2015 dataset.

Wu *et al.* proposed the feature refinement and protection network (FRPNet) for stroke lesion segmentation, which employs an encoder-decoder structure with twin attention gates to capture global and local features [33]. It minimizes feature loss in the encoder phase through a multi-dimension attention pooling method. The model provides a DSC of 60.16% in the ATLAS dataset and 85.72% in the ISLES dataset.

Li *et al.* proposed a method called BSSNet for stroke lesion segmentation . The method uses Depth-Wise Separable Convolution (DWSC) with residual connections to minimize the feature loss [34]. Multi-Scale Channel Attention (MSCA) is used to improve the segmentation accuracy. The method provides a Dice of 0.653, recall of 0.621, precision of 0.783, and IoU of 0.525.

Zhou *et al.* proposed U-Net++, which redesigns skip connections to enhance multiscale feature fusion and improve boundary localization in complex medical images. This model provided a Dice score of 0.77, demonstrating its effectiveness for lesion segmentation [35].

Ahmed *et al.* used the Transformers-Augmented MRI analysis framework that combines convolutional feature extraction with transformer attention mechanisms to provide long-range dependencies in MRI data [36]. A Dice score of 0.82 ± 0.39 on the ATLAS-2022 dataset was achieved.

Sinha *et al.* suggested EngimaNet, a DL model for stroke lesion detection and segmentation [37]. This uses Genesis-k blocks, which capture features using a dual attention mechanism and parallel convolution blocks. The accuracy of segmentation is increased by Weighted Focal Tversky-Dice (wFTD) Loss. The dice score, sensitivity, specificity, and accuracy of the model are 0.8423, 0.8452, 0.9754, and 0.9617, respectively.

Aghaei used CA to provide a parallel processing technique to minimize the temporal complexity for image analysis tasks [9]. This method takes into account the null boundary conditions, thereby addressing the complexities introduced by undefined or null values at image borders. CA based algorithm is systematically evaluated using diverse datasets with noisy images, showcasing its efficacy in detecting edges.

Kalantari *et al.* proposed brain tumor segmentation with a combination of fuzzy logic and CA [38]. The algorithm is evaluated on the BraTS 2015 dataset. This method enables segmentation without feature extraction and provides a sensitivity of 95.15% and accuracy of 99.88%.

Uguz *et al.* proposed the utilization of uniform cellular automata that use linear rules in image processing for edge detection [39]. A regular grid layout with uniform rules is applied to every cell in linear operations to improve the detection of sharp edges or transitions in pixel values. CA provides efficiency in the detection of edges with an improvement in computational time.

Chen *et al.* proposed the SLIC superpixel segmentation algorithm pertaining to image segmentation [8]. This approach involves optimization during the initialization, assignment, and update steps of SLIC to achieve more accurate and efficient superpixel segmentation. The improvements are evaluated using boundary adherence, noise sensitivity, and computational efficiency. The algorithm was tested on the BSDS 500 public dataset. The method provided a Boundary recall value of 0.838, Achievable Segmentation Accuracy of 0.972, Undersegmentation Error of 0.189, and Computational Efficiency of 0.362.

Yang *et al.* suggested an approach termed AREA-AFFINITY for predicting binding affinities in protein-protein and antibody-protein antigen interactions [7]. For protein-protein affinity prediction, AREA-AFFINITY employs 60 effective area-based models; for antibody-protein antigen binding affinity prediction, it uses 37 effective area-based models.

In comparison to the existing works, the proposed work differs in providing a multilayered approach to identify the stroke lesions. Firstly, the CA-based thresholding method extracts the brain ROI masks, which are further fine-tuned using the SLIC algorithm. Secondly, the Pixel Accuracy of the obtained ROI that are part of the lesions is improved using the antigen-antibody algorithm. Finally, the ResNet model is used to provide a diagnostic output of the image.

## 3 MATERIALS AND METHODS

In this research work, a novel technique for the detection of strokes in MRI images is proposed. The Region of Interest is extracted using threshold-based CA and superpixel segmentation with the SLIC algorithm. The Pixel Accuracy of the segmented output is improved using the antibody-antigen algorithm for accurate stroke region delineation. The delineated stroke region is trained using ResNet [10] for predictive analysis and intelligent pattern recognition. The proposed framework provides an exploratory computational approach for segmentation refinement and lesion analysis in ischemic stroke MRI. The complete architecture of the model is given in Fig. (**1**).

### 3.1 Dataset Description

A dataset from the ISLES-2015 challenge is used in this research work. The Ischemic Stroke Lesion Segmentation (ISLES) dataset is divided into Sub-acute Ischemic Stroke Lesion Segmentation (SISS) and acute Stroke outcome/Penumbra Estimation (SPES) tasks [40]. Sub-acute ischemic stroke lesion segmentations obtained from multispectral magnetic resonance imaging, which includes various sequence types such as diffusion-weighted, T1-weighted, T2-weighted, and FLAIR, are used in this work. 64 sub-acute ischemic stroke cases from the SISS task, in turn representing 64 patient datasets, are converted to 1356 2D sliced images. These 1356 2D images from the SISS task are labelled as stroke images. Also, in order to improve the stroke lesion segmentation accuracy, additional brain abnormalities are used in this work. The Brain Tumor Segmentation (BraTS) dataset is used in this work [41-43]. 1356 2D slices are extracted from the BraTS dataset, originally a part of brain tumor segmentation tasks. In the proposed work, the ISLES-2015 dataset was used as the primary source of ischemic stroke MRI images. In addition, MRI scans from the BraTS dataset were incorporated to introduce heterogeneous pathological lesion characteristics during evaluation of the proposed segmentation refinement framework. The BraTS images were included to assess the robustness of the antigen-antibody-inspired optimization strategy under varying lesion appearances. The work utilized only a publicly available dataset. As the dataset size was fixed and determined by the number of eligible cases available, no *a priori* sample size calculation or statistical power analysis was performed. The number of MRI cases included in the analysis was determined by the availability of eligible cases within the dataset.

### 3.2 Pre-processing

The generated dataset is resized to 192*192 pixels and is used in the research work. Further, to maintain experimental validity and prevent data leakage, a subject-based data split is adopted. Patient-wise partitioning ensured that slices from the same subject were not simultaneously present in both training and testing sets. To ensure that evaluation is done on entirely unseen patient data, the created dataset is split into training and testing sets at a ratio of 80% and 20%. To minimize data leakage, images allocated to the testing set were not used during model training, parameter tuning, or optimization. The training and testing datasets were maintained as independent subsets throughout the experimental procedure.

### 3.3 Thresholding-based Brain Segmentation

Segmentation of the areas in the brain is one of the most crucial procedures for diagnosis. The method involves obtaining the binary ROI mask using predetermined thresholds for brain and non-brain tissues. CA is applied to this ROI mask, gradually improving brain segmentation by fine-tuning its structure [9]. The noise in the image is removed using a four-neighborhood under Null boundary cellular automaton (FNNBCA). The best intensity value is used to replace the central cell in the neighborhood after taking into account the image pixels. Using linear CA rules that take into account the 24 neighbouring cells, the Twenty-Five Neighborhood Under Null Boundary Cellular Automaton (TFNNBCA) is employed to detect the edges. A cell’s current state and its neighbors dictate its subsequent condition. Eq. (**1**) provides the updating rule.


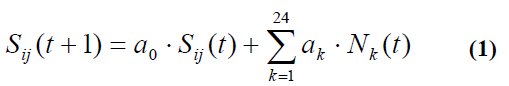


Where S*_ij_* (t+1) is the cell’s next state, S*_ij_* (t) is the cell’s current state, a_0_, a1, .. a_24_ are the coefficients of the neighbors influencing the central cell, and N*_k_*(t) represents the states of the neighboring cells.

The specified cellular automaton rule examines nearby neighborhoods and determines the expansion or contraction of pixels according to a predetermined rule. For brain extraction, an initial binary mask was generated using intensity thresholds of 0.05 and 0.95 to distinguish brain tissue from non-brain regions. The Cellular Automata (CA) refinement process employed a 5 × 5 neighborhood structure, where a pixel remained active if at least five neighboring pixels were active; otherwise, it was deactivated. This step is repeated over 10 iterations. This iterative refinement forms specific regions that correspond to different brain structures. The iteration count was empirically set to 10 to achieve effective brain-mask refinement while preserving structural continuity and reducing isolated artifacts. The largest component of the pixels obtained after the iterative refinement is the segmented brain. This segmented brain image is then subjected to further processing. The proposed CA-based brain segmentation algorithm is provided in Fig. (**2**).

### 3.4 Super Pixel Segmentation

In this research work, the brain stroke lesion is the Region of Interest (ROI). This is obtained by using the SLIC algorithm and an innovative antibody-antigen-inspired mechanism [8]. The SLIC method is effectively used to divide the 2D sliced images into smaller regions for segmentation. The SLIC method carefully moves cluster centers in a grid-like pattern throughout the image. Every cluster center acts as a superpixel’s anchor. Pixel assignment to these clusters is a crucial stage in which spatial closeness and color similarity are taken into account simultaneously. Spatial closeness ensures that the pixel closer to the superpixel center is assigned to that superpixel. This enables is providing compactness of superpixels. Color similarity is how close the color of the pixel is to the color of the central pixel. Each cluster center has a set radius around it that pixels within it are connected with, forming an initial grouping based on spatial and color similarity. The pixels with similar color or spatial proximity are grouped together. Superpixel centres are assigned across the image with cluster centres.

The proximity and color similarity are calculated to the neighnorhood pixels, which are assigned to the nearest or the most similar cluster center. Color comparison and spatial proximity are calculated by the distance metric given in eq. (**2**), which is commonly stated in the CIELAB color space.


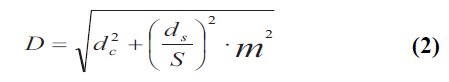


Where D is the distance metric, d_c_ is the color distance in the CIELAB color space, d_s_ is the spatial distance, S is the grid interval, and m is the compactness parameter.

This stage efficiently reduces the enormous array of pixels into initial superpixel groupings. After that, the cluster centers are regularly updated to more accurately represent the collected pixels through an iterative refinement process based on the compactness parameter. If m is small, color proximity is prioritized to determine the pixel’s distance to the superpixel center; otherwise, spatial proximity is used. The update entails calculating the average color and spatial proximity of every pixel that belongs to a specific cluster.

Iteratively refining both the spatial and color properties of the superpixels process continues until convergence. Control over the segmentation output is possible by fine-tuning parameters like the number of superpixels and the compactness. In this work, the hyperparameters such as compactness and number of superpixels are set to 10 and 200, respectively, after numerous experiments. The number of superpixels is set to 200 as the generated dataset of the preprocessed image is 192 by 192 pixels. Further, the compactness is set based on intensity in the images.

### 3.5 Antigen-antibody Optimization for Segmentation

The optimization of the ROI is performed using antigen-antibody binding affinity [7]. Immune-inspired computation is inspired by the characteristics of the human immune system. The immune system has the ability to differentiate between native and foreign cells, and it protects the body against pathogens such as bacteria, viruses, *etc*. [44]. To precisely identify, neutralize, or support the development of functional responses to an antigen from a pathogen, the immune system of the body produces B-lymphocyte cells (B cells). These B cells produce antibodies for the particular antigen. Thus, an antibody is able to identify a specific area on the antigen called an epitope. The area of the antibody that is attached to the epitope is called the paratope. Figure **3** shows the antigen-antibody recognition [45].

The functionality of an antibody depends on the affinity and specificity of an antigen. Affinity is the degree of attachment between the epitope and a paratope. The specificity of an antibody is the immune response to recognize and bind to a particular antigen. The affinity and specificity between antibodies and antigens provide the necessary immunity mechanism to the human body [46]. In this research work, the pixel with the highest intensity is the superpixel and is considered to be an antibody. Neighbouring pixels are considered antigens. The superpixel has the ability to spread to neighboring pixels, much like an antibody’s binding ability to antigens. Similar to the distinct binding features of antibodies, the algorithm carefully evaluates all the superpixels’ intensity and selects the superpixel with the highest intensity. Like the human immune response, where a particular antibody is produced based on the epitope and paratope affinity, the antigen-antibody algorithm produces binding affinity between the neighborhood pixels and the superpixel. This binding affinity is based on the non-covalent bonds between the antigen and antibody [47]. The non–covalent bonds include electrostatic bonds, hydrogen bonding, hydrophobic bonding, and van der Waals bonding [48]. This non-covalent bonding is used as the binding strength between superpixels and the neighborhood pixels. This bonding strength is determined by a threshold value. The mean intensity of the neighboring pixels is calculated and is set as the threshold value. This threshold value is used to eliminate pixels not similar to the superpixels. Thus, the superpixel binding affinity is similar to an antibody-antigen response to a pathogen. The antigen-antibody algorithm mimics the immunological response by identifying the most significant superpixels. This is performed by comparing their intensity against a predetermined threshold. Iterative refinement of the algorithm enables absorbing the neighboring pixels with similar intensity properties. The immunological phenomenon, in which antigens that replicate the specificity of an antibody, is used in the optimization algorithm to identify all the superpixels with similar features.

Similar to the immune system monitoring, the algorithm iteratively identifies the superpixels with the highest intensity and labels them as stroke lesions. The whole process is summarized in an algorithm. This novel algorithm provides an optimized segmentation result by mimicking the immune response to determine the stroke lesions on the MRI’s 2D sliced images. The algorithm for antigen-antibody binding optimization is given in Fig. (**4**).

The mean intensity I of the neighboring pixels is computed and used as the affinity threshold (α). Neighboring pixels whose intensity difference from the current superpixel (S) is less than or equal to the affinity threshold are considered to have a strong affinity and are incorporated into the evolving lesion region as given in eq. (**3**). This process is repeated iteratively until no additional neighboring pixels satisfy the affinity criterion.


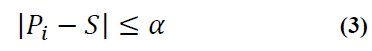


where Pi is the pixel intensity, S is the intensity of the current superpixel (antigen region), and α is the affinity threshold

### 3.6 Feature Extraction and Classification

In this proposed work ResNet-50 [8] architecture is used. For training and testing, the generated dataset with ‘stroke’ and ‘tumor’ classes is split in a ratio of 80:20. The final fully connected layer was modified to perform binary classification by mapping 2048 feature dimensions to two output classes. Additionally, the ResNet-50 model is trained using the Adam optimizer with a learning rate of 3 × 10^−5^.and CrossEntropyLoss. To achieve a < 5% error rate in recognizing the stroke lesions, the training step is repeated.

Two output classes, such as stroke and tumor images, are determined by modifying the fully connected layer in the ResNet-50 model. The model parameters are determined iteratively based on the computed loss. CrossEntropyLoss and the Adam optimizer are used to train the model. A batch size of 6 was employed during training and testing. The model was trained for a maximum of 50 epochs. To prevent unnecessary training and reduce the risk of overfitting, an early stopping criterion was adopted, whereby training was terminated when the validation accuracy exceeded 95%. Patient-wise data partitioning was used to ensure that images from the same subject were not simultaneously included in both training and testing datasets.

## 4 RESULTS AND DISCUSSION

The input brain image is subjected to CA, and a corresponding mask is obtained. This ROI is used as input for the SLIC algorithm iteratively. The intensity value of the pixels is obtained from the brain image using the extracted brain mask. The regions outside the mask are set to 0. The SLIC approach is used to create the superpixels. The pixel with the highest intensity is assigned as the superpixel. It groups areas that are close to one another and have comparable intensities. The ROI is extracted from both the thresholding and the SLIC algorithm. A superpixel is considered to be an antibody, and the neighboring pixels are an antigen. The antigens bind to the antibody based on their binding affinity (*i.e*, threshold value. Thus, the antigens acquire antibody properties, and the smaller regions bind together until no further antigens can bind. The stepwise output of a stroke image and a tumor image are shown in Figs. (**5** and **6**), respectively.

Figures **5d** and **6d** show the output of the MRI images after applying the SLIC algorithm, and Figs. (**5e** and **6e**) show the output after applying the antigen-antibody algorithm. We can infer from the above images that the pixel accuracy of the model is improved.

### 4.1 Performance Metrics

Pixel Accuracy (PA) [49] is a metric for semantic segmentation that measures the percentage of correctly classified image pixels. It is calculated as the ratio of the total number of pixels to those that are correctly classified, as described in Eq. (**4**).


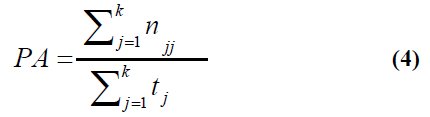


Where n_jj_ is the total number of pixels that are labelled and classified as class j, *i.e*., the total number of true positives for class j is represented by n_jj_. The total number of pixels with class j labels is denoted by t_j_.

The antigen-antibody optimization algorithm improves the pixel accuracy. 96.69% accuracy is attained after optimization. However, in lesion segmentation tasks, pixel accuracy may be influenced by class imbalance because background pixels substantially outnumber lesion pixels. Therefore, overlap-based metrics such as the Dice coefficient and IoU provide a more representative assessment of segmentation quality. The obtained Dice coefficient of 0.71 indicates promising lesion delineation performance while also highlighting opportunities for further improvement in accurately capturing lesion boundaries and smaller infarct regions. Further, the pixel accuracy after each method used is given in Table **1**. Table **2** shows the Comparative analysis of the existing works and the proposed work. Further, the proposed model yielded a precision of 0.723, recall of 0.76, F1- score of 0.71, IoU of 0.56, and Dice coefficient of 0.71.

Table **2** clearly represents a comparison of lesion segmentation performance between the proposed framework and previously reported segmentation approaches. The comparison is based on segmentation-specific evaluation metrics, including Dice coefficient and IoU. In comparison to the existing works, the proposed model provides pixel accuracy of 96.96% in the obtained ROI, with the dice score of 0.71. However, in lesion segmentation tasks, pixel accuracy may be influenced by class imbalance because background pixels substantially outnumber lesion pixels. Therefore, overlap-based metrics such as the Dice coefficient and IoU provide a more representative assessment of segmentation quality. The obtained Dice coefficient indicates promising lesion delineation performance while also highlighting opportunities for further improvement in accurately capturing lesion boundaries and smaller infarct regions. After the segmentation of the lesions, the stroke and tumor images were trained and classified using the ResNet-50 model. The accuracy of the classification was determined to be 93.09%. The experimental results indicate that the proposed antigen–antibody-inspired optimization strategy contributes to lesion-region refinement and improves segmentation consistency. These findings support the feasibility of biologically inspired optimization mechanisms for segmentation refinement within MRI-based lesion analysis pipelines.

## CONCLUSION AND FUTURE WORK

This manuscript presents a proof-of-concept framework for ischemic stroke lesion segmentation using Cellular Automata-based brain masking, SLIC superpixel segmentation, and an antigen–antibody-inspired optimization strategy. The principal contribution of the work lies in investigating the feasibility of biologically inspired optimization mechanisms for lesion-region refinement in MRI images. The system incorporates ResNet-50 in order to recognize features that are essential for stroke diagnosis. This comprehensive model enables effective stroke lesion identification by extracting the ROI for deep learning classification. Thus, this method facilitates lesion identification and segmentation, which may support imaging assessment during early stroke evaluation. Early and accurate diagnosis has significant consequences in the field of healthcare. The model achieved pixel accuracy, classification accuracy, precision, recall, F1-score, IoU, and Dice coefficient of 96.96%, 93.09%, 0.723, 0.76, 0.71, 0.56, and 0.71, respectively. The proposed framework achieved promising lesion segmentation performance, with a Dice coefficient indicating substantial agreement between predicted and reference lesion regions. While a high pixel accuracy was obtained, overlap-based metrics provide a more reliable assessment of segmentation quality in the presence of class imbalance. The proposed framework demonstrated promising performance for lesion segmentation refinement across heterogeneous pathological MRI datasets.

Although promising segmentation performance was achieved on the evaluated dataset, the absence of external validation limits conclusions regarding robustness and generalizability. Future work will focus on evaluating the proposed framework on independent datasets across diverse imaging conditions and patient populations. The proposed framework demonstrated promising segmentation performance and should be interpreted as a methodological and exploratory investigation rather than evidence of clinical deployment readiness. Further, the BraTS dataset used in the dataset was not intended to represent clinically representative non-stroke cases. Instead, it was incorporated to introduce pathological heterogeneity and evaluate the robustness of the proposed antigen–antibody-inspired segmentation refinement strategy. A limitation of the present study is the relatively small number of patients available for evaluation. While multiple image slices were extracted from each subject, these samples are not fully independent and may introduce a risk of overfitting. Also, Variations in MRI acquisition protocols, scanner characteristics, lesion appearance, and patient demographics may influence model performance when applied to independent cohorts. Therefore, further validation using larger patient cohorts and external datasets is necessary to establish the robustness and generalizability of the proposed framework. Although Dice coefficient and IoU were used to evaluate segmentation performance, boundary-based metrics such as Hausdorff Distance (HD) and Average Symmetric Surface Distance (ASSD) were not included in the current study. These metrics will be incorporated in future evaluations. Statistical significance testing was also not performed because the present work focused on evaluating the proposed framework on a single experimental configuration. Also, future work will investigate cross-validation-based evaluation protocols to further assess model stability and robustness.

## Figures and Tables

**Fig. (1):**
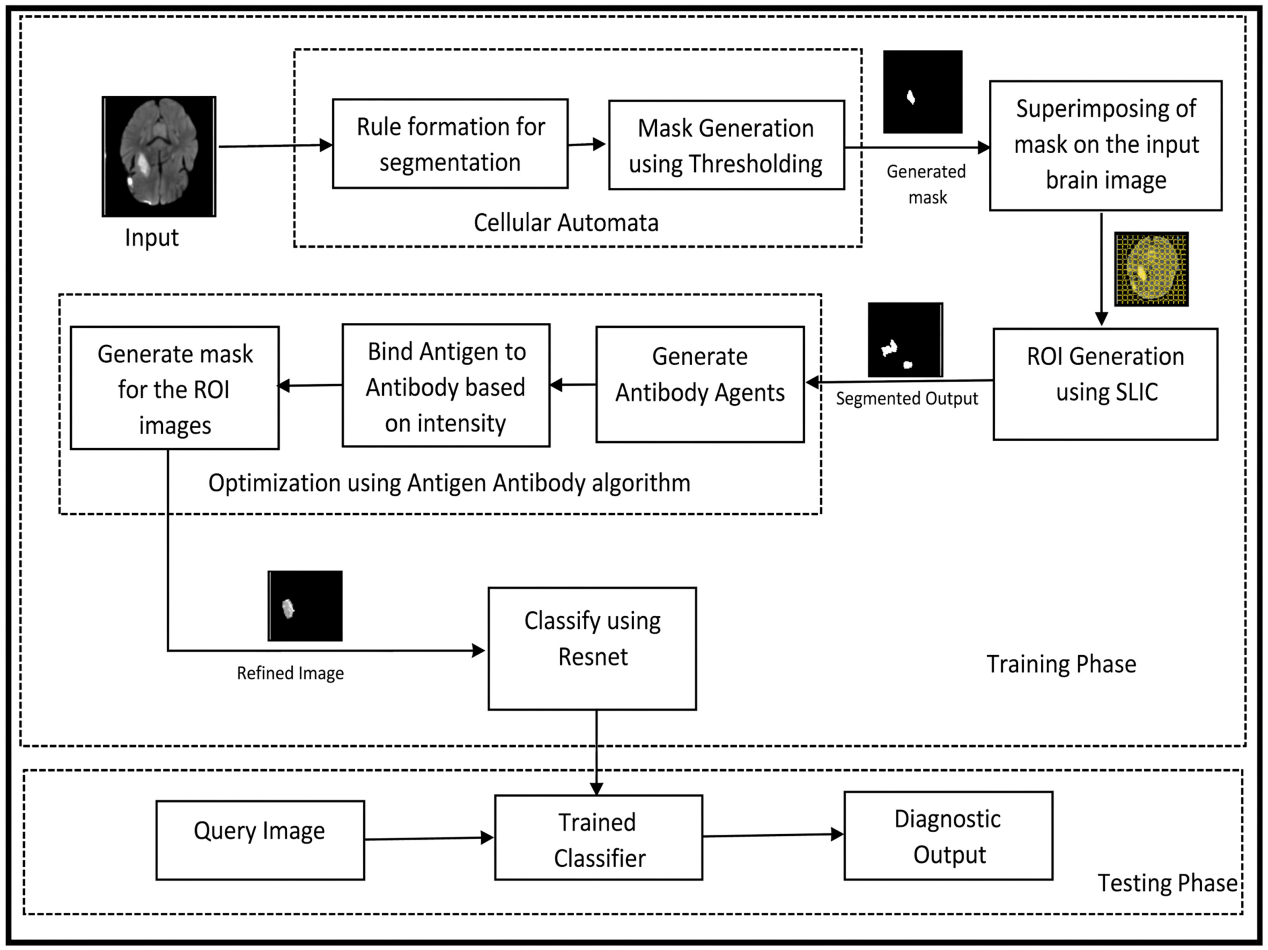
Overall architecture.

**Fig. (2):**
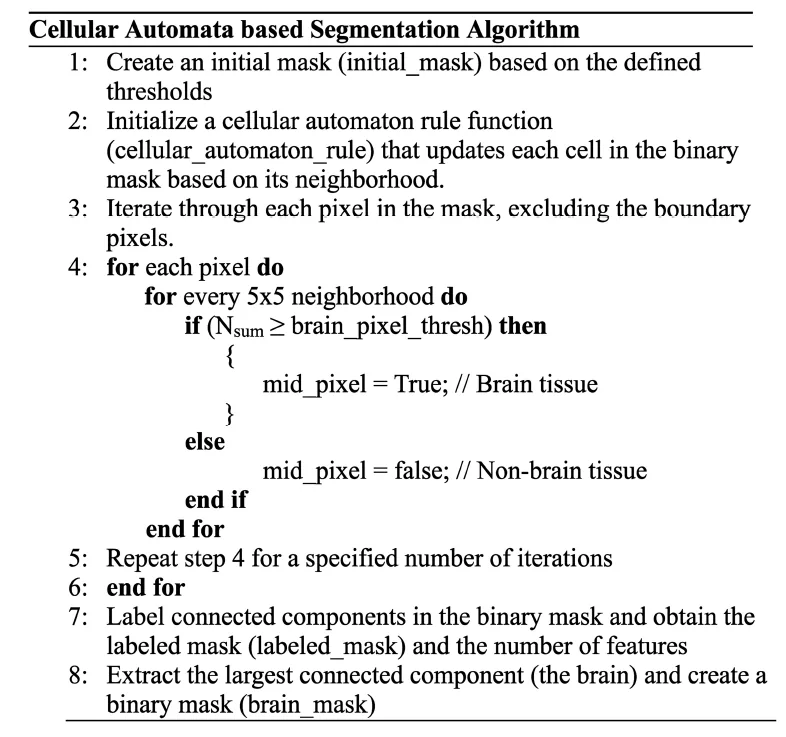
Proposed CA-based segmentation algorithm.

**Fig. (3):**
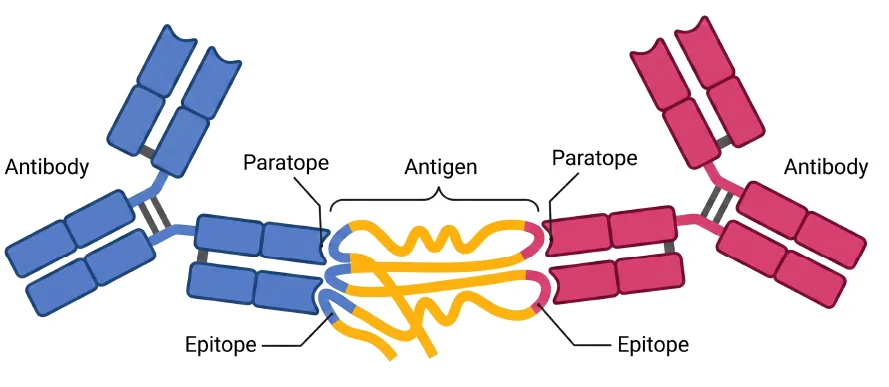
Antigen-antibody recognition. Reproduced from Bukhari SNH, Jain A, Haq E, Mehbodniya A, Webber J. Machine learning techniques for the prediction of B-cell and T-cell epitopes as potential vaccine targets with a specific focus on SARS-CoV-2 pathogen: A review. Pathogens. 2022;11(2):146, under the Creative Commons Attribution 4.0 International (CC BY 4.0) License [45].

**Fig. (4):**
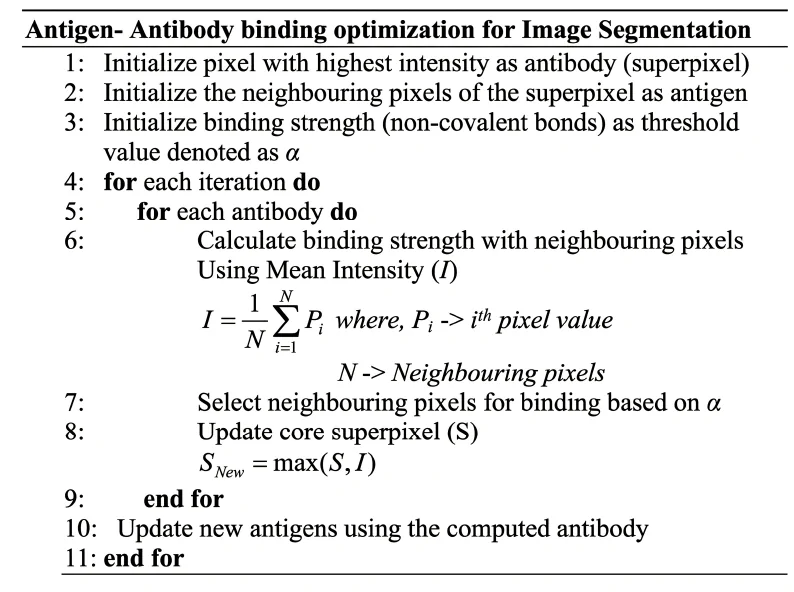
Antigen-antibody binding optimization for image segmentation.

**Fig. (5):**
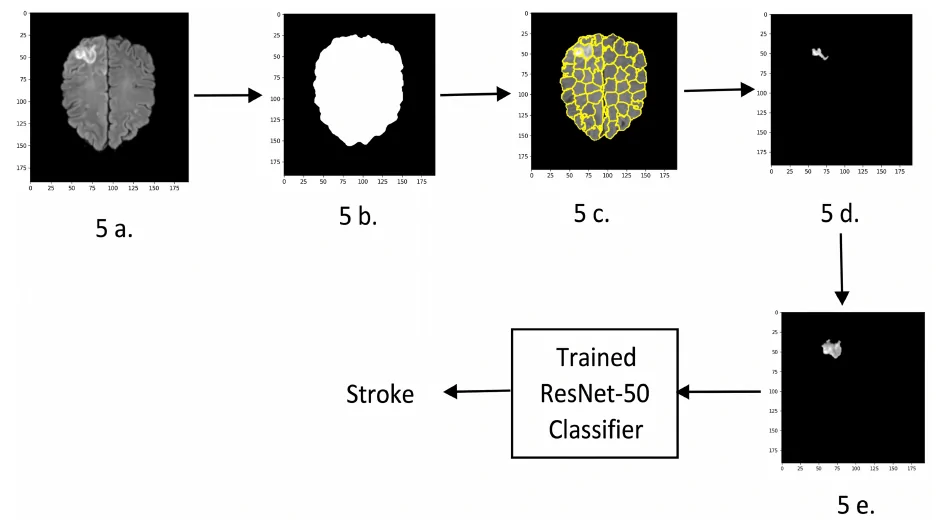
Stepwise output of stroke image. (**a**). Input image. (**b**). Mask generated after CA. (**c**). Applying SLIC algorithm. (**d**). ROI obtained after SLIC algorithm. (**e**). ROI obtained after antigen- antibody algorithm.

**Fig. (6):**
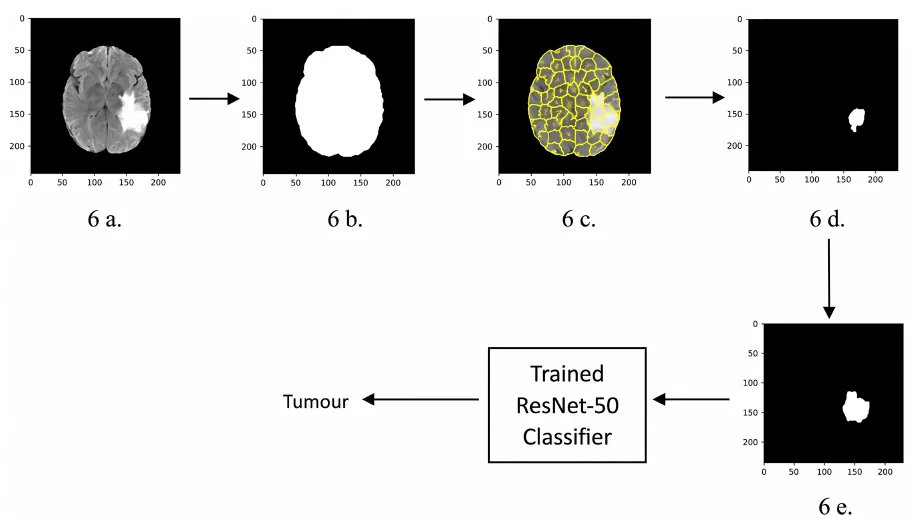
Stepwise output of tumor image. (**a**). Input image. (**b**). Mask generated after CA. (**c**). Applying the SLIC algorithm. (**d**). ROI obtained after the SLIC algorithm. (**e**). ROI obtained after antigen- antibody algorithm.

**Table 1:** Pixel accuracy after using various methods.

**Method Used**	**Pixel Accuracy**
Using Thresholding with CA (A)	67.27
Using Super Pixel Integration (B)	87
Optimizing using Antibody Antigen Algorithm (C)	96.96

**Table 2:** Comparative analysis of the existing works and the proposed work.

**Method Used**	**Pixel Accuracy**	**Precision**	**Recall**	**Dice Score**
FCN-8 [26]	65.95	0.488	0.4531	0.672
3D Unet [27]	64.66	0.68	0.60	0.58
UNet++ [35]	87.32	0.701	0.69	0.77
Transformers-Augmented MRI Analysis [36]	88.45	0.73	0.70	0.82
EngimaNet Model [37]	95.42	0.70	0.67	0.82
Proposed Model (A+B+C)	**96.96**	**0.723**	**0.76**	0.71

## Data Availability

The datasets used in this study are publicly available and can be accessed from the respective repositories listed below: 1. ISLES Dataset (Ischemic Stroke Lesion Segmentation Challenge): The ISLES dataset used in this study is publicly available for research purposes through the official challenge website and associated repositories. The dataset can be accessed through the following link: https://www.isles-chal lenge.org/. 2. BraTS Dataset (Brain Tumor Segmentation Challenge): The BraTS dataset used in this study is publicly available for academic and research use through the official BraTS challenge platforms. The dataset can be accessed at https://www.med.upenn.edu/cbica/brats2020/data.html

## LIST OF ABBREVIATIONS

MRI: Magnetic Resonance Imaging
CA: Cellular Automata
SLIC: Simple Linear Iterative Clustering
ISLES: Ischemic Stroke Lesion Segmentation
ResNet: Residual Network
IoU: Intersection Over Union
NIHSS: NIH Stroke Scale/Score
CT: Computed Tomography
PET: Positron Emission Tomography
fMRI: Functional MRI
ROI: Region of Interest
ML: Machine Learning
DL: Deep Learning
CAD: Computer-Aided Diagnosis
CBF: Cerebral Blood Flow
CBV: Cerebral Blood Volume
MTT: Mean Transit Time
Tmax: Time Of Maximum Value Of Residue
MAE: Mean Absolute Error
PSNR: Peak Signal-to-Noise Ratio
SSIM: Structural Similarity Index Measure
PCC: Pearson Correlation Coefficient
CTP: CT Perfusion
RF: Random Forest
SVM: Support Vector Machine
DT: Decision Tree
NCCT: Non-Contrast CT
ELV: Expected Label Value
FCN: Fully Convolutional Network
ECG: Electrocardiography
EEG: Electroencephalography
AUC: Area Under the Curve
CNN: Convolutional Neural Networks
LSTM: Long Short-Term Memory
HCSNet: Hybrid Contextual Semantic Network
FRPNet: Feature Refinement And Protection Network
DWSC: Depth-Wise Separable Convolution
MSCA: Multi-Scale Channel Attention
wFTD: Weighted Focal Tversky-Dice
SISS: Stroke Lesion Segmentation
SPES: Stroke Penumbra Estimation
BraTS: Brain Tumour Segmentation
TFNNBCA: Twenty-Five Neighbourhood Under Null Boundary Cellular Automaton
B cells: B-Lymphocyte Cells
2D: Two Dimension
PA: Pixel Accuracy
HD: Hausdorff Distance
ASSD: Average Symmetric Surface Distance
